# Unusual Lesions in a Long‐Term Dialysis Patient

**DOI:** 10.1111/odi.70230

**Published:** 2026-07-09

**Authors:** Domenico De Falco, Giuseppe Ingravallo, Massimo Petruzzi

**Affiliations:** ^1^ Interdisciplinary Department of Medicine University of Bari “Aldo Moro” Bari Italy; ^2^ Department of Emergency and Transplantation, Pathology Section University of Bari Medical School Bari Italy

## 1 Case Report

A 67‐year‐old man presented to our attention due to the onset of extensive cutaneous and lingual nodular lesions for about a month. The patient had a medical history of chronic renal failure for 30 years and osteoarthritis; and had been on dialysis for about 11 years.

On physical examination, the cutaneous lesions appeared circumscribed, non‐tender to palpation, and had a hard and rough consistency. These lesions were multiple and located on the flexor surfaces of the upper limbs and on the patient's back, corresponding to the thoracolumbar fascia and the infraspinatus fascia. During the intraoral examination, clusters of non‐tender, hard, yellowish nodular lesions were observed on the dorsal surface of the tongue (Figure 1). Additionally, the patient did not exhibit any general symptoms that could suggest ongoing infections. The patient's blood tests showed a significant increase in creatinine, blood urea nitrogen, and beta‐2 microglobulin. Specifically, the beta‐2 microglobulin levels reached 25 mg/L.

## 2 What Is Your Diagnosis?

Based on the patient's history, physical examination, and laboratory findings, which one of the following is the most suspicious diagnosis?

- Tubercolosis
- Sarcoidosis
- Amyloidosis
- Lipomatosis

**FIGURE 1:**
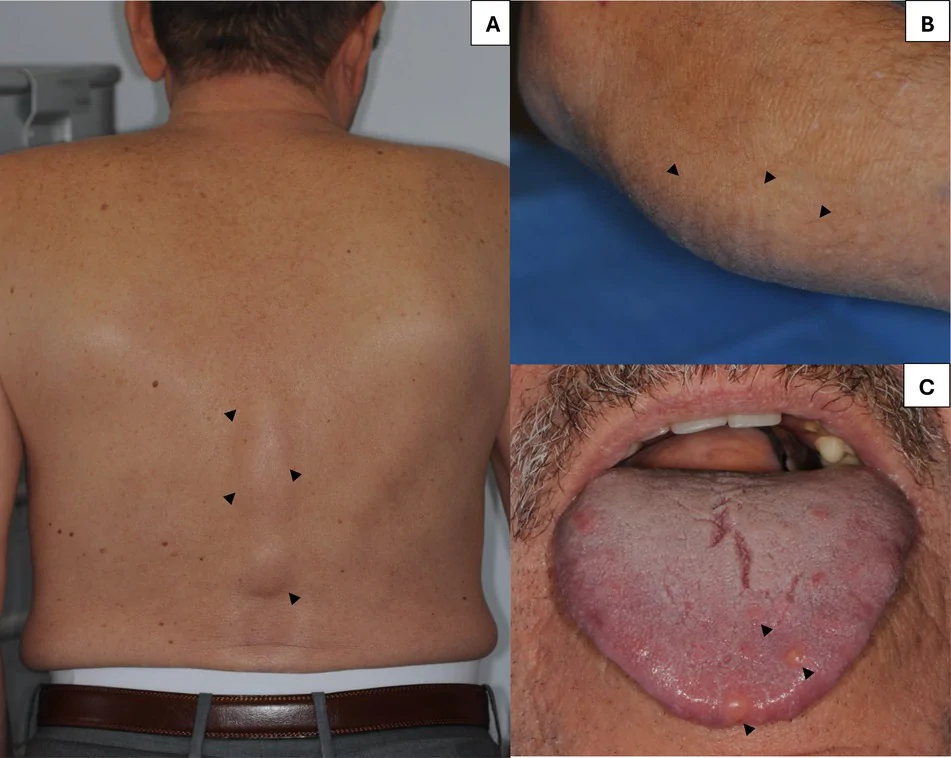
(A, B) Hard, persistent nodules on the flexor surface of the limbs and along the midline of the back; (C) multiple nodular lesions on the dorsal surface of the tongue.

## 3 Diagnosis

The clinical findings, together with the pathological features, support the diagnosis of dialysis‐related amyloidosis (DRA). The patient had been recommended to undergo an excisional biopsy of the nodular lesions present on the dorsal surface of the tongue. Two tongue lesions were excised and subjected to pathological examination. The biopsy examination revealed the presence of eosinophilic amyloid material in the papillary dermis, positive for Congo Red staining with apple‐green birefringence under polarized light (Figure 2). Immunohistochemistry confirmed the presence of beta‐2 microglobulin. DRA is a well‐recognized long‐term complication of hemodialysis, resulting from the accumulation and tissue deposition of β2‐microglobulin (Simão Dos Santos et al. 2013). This condition is closely associated with the use of low‐flux cellulose dialysis membranes, which promote the tissue deposition of beta‐2 microglobulin in the form of amyloid (Simão Dos Santos et al. 2013; Yusa et al. 2001; Matsuo et al. 1997). DRA typically affects the bones and joints, with a high prevalence in patients undergoing prolonged dialysis treatment. In patients with more than 15 years of dialysis, systemic manifestations may occur, including extra‐articular involvement such as cutaneous and oral tissues (Simão Dos Santos et al. 2013). Lingual involvement is rare, and only a few cases have been reported in the literature (Simão Dos Santos et al. 2013). Clinically, lingual lesions present as firm, yellowish nodules, most commonly located on the lateral borders of the tongue. Lingual DRA may lead to reduced tongue mobility, speech articulation disorders, or taste disturbances, particularly in cases with more extensive involvement (Yusa et al. 2001). Diagnosis is confirmed by pathological exam, revealing the presence of β2‐microglobulin deposits (Simão Dos Santos et al. 2013; Matsuo et al. 1997). These deposits stain positively with Congo red and exhibit apple‐green birefringence under polarized light, a hallmark of amyloid material (Matsuo et al. 1997). Once DRA is diagnosed, management is largely supportive and focuses on reducing the ongoing β2‐microglobulin burden while addressing local symptoms (Portales‐Castillo et al. 2020). Practical measures include optimizing the dialysis prescription to enhance clearance of middle molecules, for example, using high‐flux, biocompatible membranes and ensuring strict use of ultrapure dialysate, and considering convective strategies such as online hemodiafiltration when available (Portales‐Castillo et al. 2020). Kidney transplantation should be considered in eligible patients, as it can halt further β2‐microglobulin accumulation and promote clinical stabilization (Portales‐Castillo et al. 2020). Symptom control with appropriate analgesia and conservative measures (physiotherapy and splinting) helps preserve joint function (Portales‐Castillo et al. 2020). When lesions substantially limit limb use or cause compressive complications, surgical or orthopedic intervention may be indicated (Portales‐Castillo et al. 2020). In selected settings, β2‐microglobulin adsorption approaches have also been explored as adjunctive strategies, primarily with the aim of improving symptoms (Portales‐Castillo et al. 2020).

## 4 Outcome

The patient was directed toward a multidisciplinary management approach, involving Nephrologists for the evaluation of dialysis permeability modulation and corticosteroid therapy for managing osteoarticular features.

**FIGURE 2:**
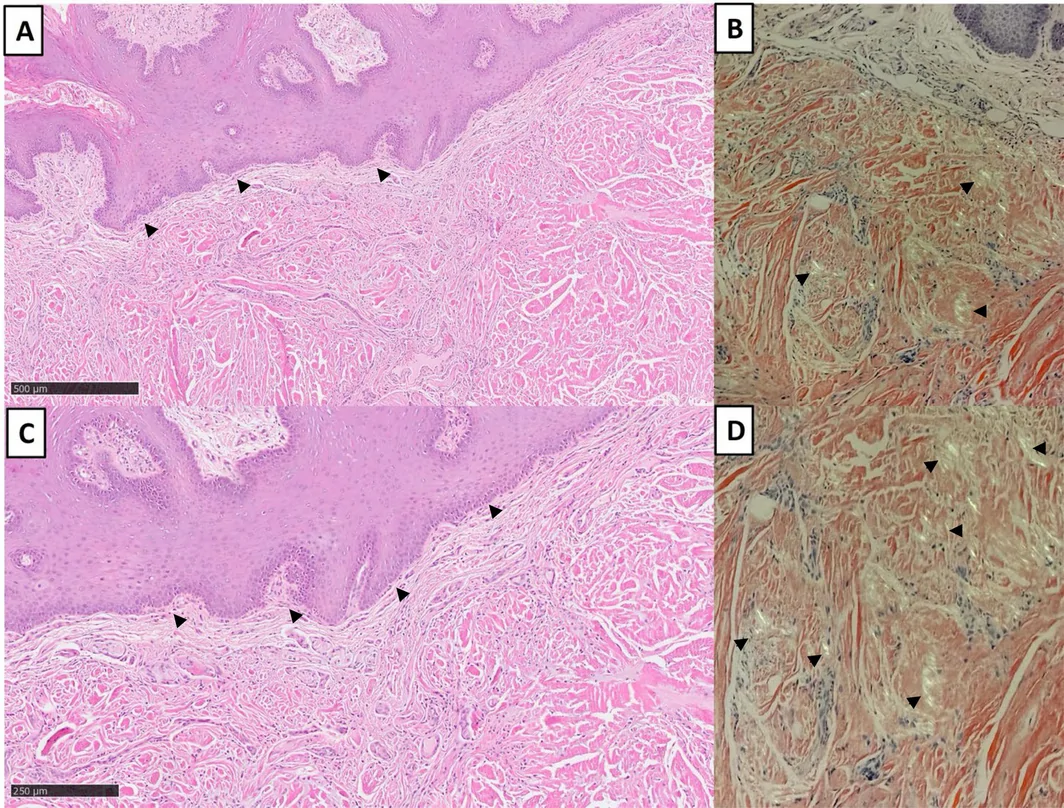
(A) Hematoxylin and eosin staining showing deposition of amorphous, eosinophilic material within the lamina propria/submucosa (arrows), suggestive of amyloid. Scale bar: 500 μm. (B) Congo red staining of the same specimen examined under polarized light, highlighting amyloid deposits (arrows). (C) Hematoxylin and eosin staining (higher‐power view of panel A) demonstrating the amorphous eosinophilic deposits within the lamina propria/submucosa in greater detail (arrows). Scale bar: 250 μm. (D) Congo red staining under polarized light (higher‐power view of panel B) showing apple‐green birefringence in the indicated areas (arrows), consistent with amyloid deposition.

## Author Contributions

**Domenico De Falco:** methodology, formal analysis, writing – original draft, data curation. **Giuseppe Ingravallo:** conceptualization, methodology, writing – review and editing, supervision. **Massimo Petruzzi:** conceptualization, methodology, supervision, writing – review and editing.

## Consent

The patient reported in this manuscript provided written informed consent for the publication of the case details.

## Conflicts of Interest

All authors have no conflicts of interest to disclose.

## Data Availability

The data that support the findings of this study are available from the corresponding author upon reasonable request.
